# Aromatherapy and Essential Oils: Holistic Strategies in Complementary and Alternative Medicine for Integral Wellbeing

**DOI:** 10.3390/plants14030400

**Published:** 2025-01-29

**Authors:** Karina Caballero-Gallardo, Patricia Quintero-Rincón, Jesus Olivero-Verbel

**Affiliations:** 1 Functional Toxicology Group, School of Pharmaceutical Sciences, Zaragocilla Campus, University of Cartagena, Cartagena 130014, Colombia; patriciaquintero@gmail.com; 2 Environmental and Computational Chemistry Group, School of Pharmaceutical Sciences, Zaragocilla Campus, University of Cartagena, Cartagena 130014, Colombia; joliverov@unicartagena.edu.co; 3 Research Group Design and Formulation of Medicines, Cosmetics, and Related, Faculty of Pharmaceutical and Food Sciences, University of Antioquia, Medellin 050010, Colombia

**Keywords:** aromatic plants, essential oils, holistic wellness, complementary, alternative medicine, healthcare settings

## Abstract

Complementary and alternative medicine (CAM) encompasses a variety of ancient therapies with origins in cultures such as those of China, Egypt, Greece, Iran, India, and Rome. The National Institutes of Health (NIH) classifies these integrative therapies into five categories: (1) mind–body therapies, (2) biological practices, (3) manipulative and body practices, (4) energy medicine, and (5) whole medical systems, including traditional Chinese medicine and Ayurvedic medicine. This review explores the role of biological practices utilizing aromatic plants, particularly through inhalation aromatherapy and massage with essential oils, as effective complementary strategies within health systems. The review compiles information on the most commonly used plants and essential oils for holistic health maintenance from a complementary and alternative perspective. Given their accessibility and relative safety compared to conventional treatments, these therapies have gained popularity worldwide. Furthermore, the integration of essential oils has been shown to alleviate various psychological and physiological symptoms, including anxiety, depression, fatigue, sleep disorders, neuropathic pain, nausea, and menopausal symptoms. Among the studied plants, lavender has emerged as being particularly notable due to its broad spectrum of therapeutic effects and its designation by the US Food and Drug Administration (FDA) as “Generally Recognized as Safe”. Other essential oils under investigation include eucalyptus, damask rose, sandalwood, vetiver, calamus, frankincense, chamomile, lemon, grapefruit, tangerine, orange, sage, rosemary, garlic, and black pepper. This study emphasizes the potential benefits of these aromatic plants in enhancing patient well-being. Additionally, it underscores the importance of conducting further research to ensure the safety and efficacy of these therapies.

## 1. Introduction

Traditional practices based on aromatic plants have been a valuable resource for treating human emotional and physical ailments for centuries. These plants are a rich source of potential compounds for the treatment of various pathologies and have made it possible to enrich the plethora of pharmacological compounds used in conventional medicine; in fact, they continue to be a promising resource for the development of new drugs [1]. Nowadays, new therapeutic approaches such as the application of nanotechnology for the diagnosis, treatment, and prevention of diseases [2], drugs targeting receptors, surface antigens, and signaling pathways [3], tissue regeneration therapy based on knowledge of pluripotent stem cells [4], and digital therapy using artificial intelligence [5] are part of the strategies that have been developed due to the advancement of science and technology. However, despite these advances, traditional practices based on the use of herbal extracts and aromatherapy-essential oils (AEOs) as a complementary or alternative therapy to Western medicine have gained particular interest in professional care systems around the world, supported by evidence-based practices [6], becoming a fundamental tool to achieve the psychological and physiological well-being of patients, improving their quality of life by reducing their discomfort effectively, economically, and with fewer adverse effects compared to conventional treatments [7,8,9].

Gnatta et al. [10] stablish a clear distinction between complementary and alternative treatments concerning Western medicine. In this context, the use of aromatic plants, and especially their essential oils (EOs), is considered a complementary therapy when integrated with conventional Western medicine treatments to improve the patient’s well-being. On the other hand, alternative therapies are characterized by completely replacing conventional medical care. This therapeutic strategy is justified because studies have demonstrated that somatic disorders and distressing behaviors could be improved by using extracts, especially EOs. Therefore, aromatherapy has gained great popularity as a complementary therapy for treating physical and emotional diseases and promoting holistic health [11]. Some authors consider aromatherapy a science since rigorous methodologies have been established to confirm the therapeutic properties of these natural products [12]. Among the therapeutic benefits studied are listed the improvement of mood alterations [13], anxiety disorders [14], depression [15], insomnia [16], fatigue [17], chronic pain [18], restless legs syndrome [19], migraine [20], and behavioral disturbances in dementia [21], as well as improved somatic symptoms, such as blood pressure and respiratory rate, among others [11]. This discipline emerged as an alternative for handling many medical conditions and currently a global market growth of $5 trillion by 2050 has been estimated [22].

Complementary and alternative medicine (CAM) includes ancient practices, including inhalation aromatherapy, massage, homeopathy, chiropractic, acupuncture, and Ayurvedic medicine, among others, which originated in countries such as Iran, China, Rome, Greece, Egypt, and India [15]. These civilizations discovered that extracts obtained from plant parts such as flowers, bark, roots, seeds, petals, and leaves, among others, improved the mind–body state of individuals [23]. These extracts were used during World War I and II for treating gangrenous wounds, burns, and other trauma as well as for treating mental disorders in society that arose as consequences of war [24]. CAM is popular because people have the perception that natural products cause fewer side effects and are less dangerous or less toxic. The use of CAM is closely related to pleasant sensations because it helps to establish physical and emotional balance [23]. In this sense, people feel greater holistic well-being and a strengthened immune system [25]. Today, CAM approach is a fundamental tool for health care units. CAM is part of the strategies used by nurses to alleviate the ailments of patients, besides being a simple method that relieves stress in these professionals [26].

Information presented in this review was collected using electronic searchers for articles published in peer-reviewed journals (Google Scholar, PubMed, Springer, Scopus, and Science Direct). The search terms were “Aromatic plants” or “Essential oils” or “Complementary and Alternative Medicine” or “Aromatherapy” or “Biological activities” or “Therapeutic effects”. Relevant scientific research in the area of interest and crucial reference articles were used as inclusion criteria. Duplicate publications and irrelevant articles were excluded.

## 2. Aromatherapy and Essential Oils in Complementary and Alternative Medicine

**Figure 1:**
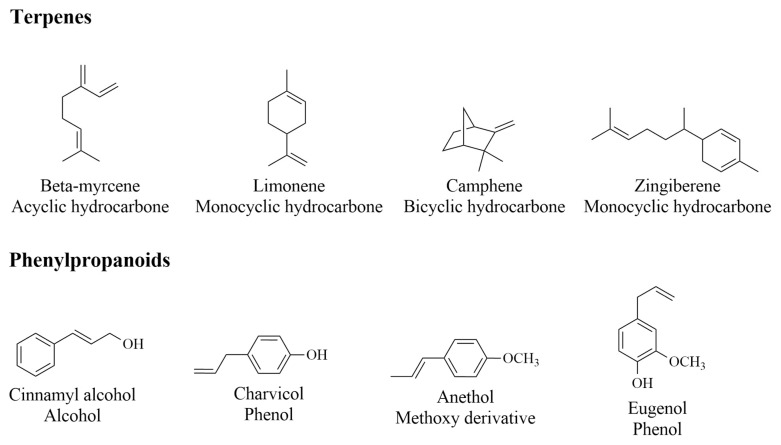
Chemical structures of terpenes and phenylpropanoids found in EOs of plants.

Aromatherapy is a complementary health strategy that uses EOs in order to improve the integral health of individuals [10]. The term was coined by the French chemist Rene-Maurice Gattefosse in the 1920s. This strategy is carried out through the use of EOs as a tool for therapeutics to promote body, mind, and spirit health [23,27]. EOs are liquid extractives obtained from herbs that are marketed as pure extracts to relieve psychosomatic disorders through the stimulation of the olfactory system [28]. EOs consist of complex mixtures of phytoconstituents with a wide range of molecular arrangements produced by mevalonate, methylerythritol, and shikimic acid biosynthetic pathways. Their structures are chemically grouped as terpenes (biosynthesized via the mevalonate acid and methylerythritol phosphate (MEP) pathways) or phenylpropanoid classes (biosynthesized via the shikimic acid pathway) [29]. Some chemical structures of terpenes and phenylpropanoids from EOs are shown in Figure 1.

Essential oils have a low molecular weight, high refractive index, lower density than water [29], and are also lipophilic and highly volatile under normal conditions [30]. They are extracted from the bark of plants, roots, leaves, flower petals, or stems by hydro-distillation, solvent extraction, supercritical fluid extraction, and solvent-free microwave-assisted extraction [27]. EOs have a wide range of applications in aromatherapy, traditional medicines, Chinese and alternative medicines, massage therapies, cosmetics, perfumes, and food industries [31].

The literature indicates that aromatherapy is a pivotal strategy in various areas of health care, including surgical medical care, geriatrics, mental health, obstetrics, and pediatrics. Its use is popular in dialysis sessions to reduce neuropathic pain in kidney patients [23]. It is also very useful to relieve vascular complications induced by diabetes mellitus [32], treat symptoms in neurovascular dysfunction diseases [20], to provide relief from psychosomatic discomfort during the puerperium [24], to reduce symptoms in cardiovascular diseases [33], to treat neurodegenerative manifestations and age-related progressive deterioration [34], to treat discomfort in oncology patients [35], to relieve menopausal symptoms [36], to relieve restless leg syndrome [19], and for the treatment of both respiratory infections [37] and skin infections [25].

*Molecular Mechanisms of Essential Oils and Phytochemical*

Research on aromatherapy has shown that plant odors have a significant effect on emotions in humans [13]. This is because the stimulation of the olfactory system with essential oils modulates the activities controlled by the Autonomic Nervous System (ANS). For example, lavender EO (*Lavandula angustifolia*, Lamiaceae) has been reported to promote appetite by stimulating parasympathetic nerves and pomelo or grapefruit oil (*Citrus paradisi*, Rutaceae) reduces appetite through sympathetic nerve stimulation [38].

**Figure 2:**
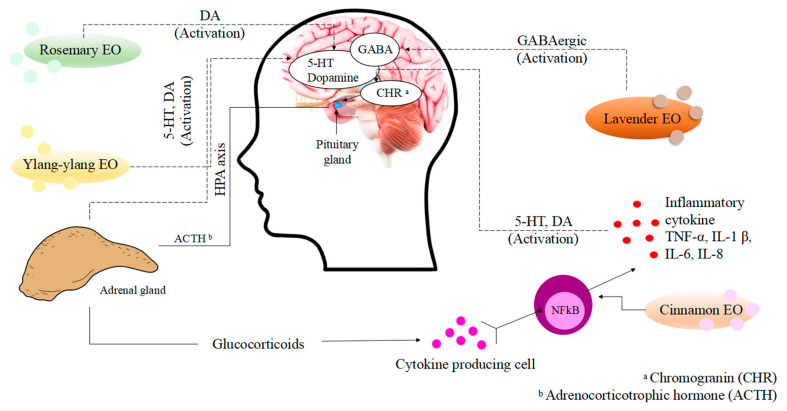
Mechanism actions of essential oils on the Central Nervous System. Adapted from Soares et al. [40].

Studies have suggested that some EOs exert an anxiolytic effect due to the ability of these liquid extracts to modulate the GABAergic system and sodium channels, but also due to their ability to target transient receptor potential channels. EO use has also been linked to decreased pro-inflammatory and anti-inflammatory responses to stress. In general, it has been suggested that EOs act on the Central Nervous System (CNS) through the activation of various components of the brain. In this sense, lavender EO exerts an anxiolytic anti-stress effect, improves mood, is analgesic, and relieves pain because it activates the GABAergic system, reducing the excitability of peripheral and central nerves. The inhibition of voltage-gated calcium channels reduces serotonergic 5-HT1A receptor activity. Increases in parasympathetic tone are also part of the mechanisms that describe the biological activity of this AE. Ylang-ylang EO exerts its relaxing and antidepressant properties because it activates the Autonomic Nervous System (ANS) and affects the DAergic system, increases serotonin (5-HT) levels, and decreases glucocorticoid levels. Additionally, this oil affects the hypothalamic–pituitary–adrenal (HPA) axis by lowering glucocorticoid levels to produce a calming effect. In vivo studies have shown that this EO exerts an anxiolytic effect in male mice because it promotes a decrease in dopamine (DA) in the striatum, as well as a reduction in the concentration of 5-HT in the hippocampus. Furthermore, ylang-ylang EO decreases the 5-hydroxyindole acetic acid (5-HIAA)/5-HT ratio. Rosemary EO enhances the activation and secretion of DA, which explains the anxiolytic effect and the improvement of cognitive function. Finally, the anxiolytic effect of cinnamon oil is due to a downregulation of nuclear factor kappa-light-chain-enhancer, NFкB [39,40]. The biological results of lavender, rosemary, cinnamon, and ylang-ylang in the CNS are shown in Figure 2.

**Figure 3:**
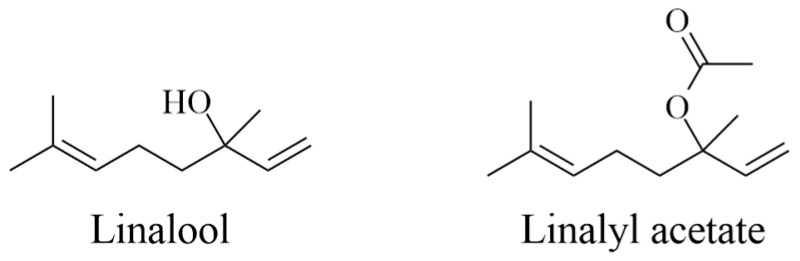
Chemical structures of major phytoconstituents from lavender: linalool and linalyl acetate.

These mechanisms of action have also been proposed to explain the antinociceptive and anticonvulsant effects of EOs [40]. EOs obtained from lavender, lemon (*Citrus limon*), sandalwood (*Santalum album*, Santalaceae), orange (*Citrus sinensis*, Rutaceae), Damask rose (*Rosa damascena*, Rosaceae), clary sage (*Salvia sclarea*, Lamiaceae), bergamot (*Citrus bergamia*, Rutaceae), Roman chamomile (*Anthemis nobilis*, Asteraceae), ylang-ylang (*Cananga odorata*, Annonaceae), and *Boswellia* and *Pelargonium* species are extensively used in aromatherapy because they have a strong anxiolytic property [31,40,41]. Some phytoconstituents have also shown therapeutic effects. In this sense, linalool and linalyl acetate (Figure 3) obtained from lavender EO are anxiolytic because both compounds inhibit voltage-gated calcium channels, reduce 5HT1A receptor activity, and increase parasympathetic tone [42].

Studies have shown that phytoconstituents of EOs can reach the blood, cross the blood–brain barrier and enter the CNS following oral administration, inhalation aromatherapy (direct inhalation with a tissue or cotton ball infused with drops of essence, room spray, lamp diffusion, or nasal inhaler), dermal application (massotherapy), and subcutaneous or intraperitoneal injection [11,43]. This indicates that the EOs or phytoconstituents used in massages or inhalation aromatherapy are absorbed through the skin or the olfactory system, respectively. Other studies indicate that EOs reach the limbic system and thalamus, stimulating the production of chemical compounds that reduce stress levels and enhance healing rates [44].

EOs or aromatic plants used in massages are gradually absorbed through the skin (10 to 40 min after the application). However, it is well known that the positive effects on the brain, the limbic system, and the thalamus are due to the stimulation that EOs induce on nasal epithelium receptor cells, leading to the release of endorphins and serotonin [45,46]. For example, lavender EO or the linalyl acetate and linalool found in this plant stimulate the parasympathetic system. These phytoconstituents exert modulator properties on mood states. Moreover, these can be used as an effective strategy against hemodynamic instabilities and could be an effective treatment for physiological and psychological disorders, among them being fatigue, depression, distress, blood pressure, pain, pruritus, nausea, and vomiting [6,7,46]. Studies have demonstrated the absorption properties of volatile compounds such as linalool and linalyl in the bloodstream during massage [9], and urine and respiration have been proposed as routes of elimination [47].

In general, the physiological and psychological effects of EOs appear by exposition of these on organs of the body, stimulating the olfactory system through the sense of smell. Volatile molecules pass through the nasal passages and stimulate nerve signal transmission through the olfactory bulb, interacting with olfactory nerve receptors and also activating the limbic system and cerebellar cortex [48]. Both the amygdala and hippocampus are structures in the limbic system that process the signals induced by EOs. When EOs are absorbed through the skin or inhaled, these stimulate the amygdala and hippocampus to trigger effects on physical, emotional, and mental health. For this reason, emotional reactions to aromas are governed by the amygdala and olfactory memory by the hippocampus [49].

*Lavandula angustifolia* is the aromatic plant most popular in CAM. Lavender EO is used in inhalation aromatherapy or massage to stimulate blood circulation, affecting heart function. It has antidepressant, antispasmodic, and antibacterial properties and is both a relaxant and a pain reliever. Its use extends to treating migraines and insomnia [50]. The sedative and narcotic effects of lavender are due to linalool and linalyl acetate, respectively. Linalool acts on γ-amino-butyric-acid receptors in the CNS, while linalyl acetate exerts a narcotic function [48]. Lavender oil aromatherapy has gained recognition for its therapeutic benefits, particularly in reducing stress and improving sleep quality among patients undergoing surgical procedures. Research indicates that the inhalation of lavender essential oil can lead to significant physiological improvements, including normalizing vital signs such as systolic blood pressure, respiratory rate, oxygen saturation, and heart rate [51].

Inhalation of lavender EO calms patients with cardiac surgery and improves physiological indicators by reducing cortisol secretion, decreasing sympathetic system activity, and increasing parasympathetic system activity. Lavender inhibits the release of acetylcholine and increases melatonin (sleep hormone) levels, while linalool acetate can relax the smooth muscles of arteries and interacts with serotonin transporters and monoamine oxidase A (MAO-A), N-methyl-D-aspartate (NMDA) and γ-aminobutyric acid-A (GABA-A) receptors [51,52,53]. The FDA conferred lavender EO the status of “Generally Recognized as Safe”; therefore, this EO can be used as a dietary supplement [54].

## 3. Therapeutic Applications of Essential Oils

Currently, many routine beauty and personal care products include EOs as part of their formulation. In fact, the use of these extracts has gained popularity in health care systems. Despite this progressive growth in the use of EO as a complementary treatment to achieve the holistic health of individuals, the effects of chronic exposure to the ingredients on human health have been little studied. The FDA classifies EOs for aromatherapy as cosmetics because, according to its guidelines, EOs are not drugs for treating or preventing diseases [22]. However, EOs have been proposed to act as a drug or enzyme (systemic effect), although the ability to modulate mood has been linked to affective effects or “reflective” effects, and even a placebo effect has been proposed [25].

Over the years, EOs have shown variability in therapeutic effects, which is related to the unique chemical complexity that characterizes each extract. Due to the side effects reported in the literature and the complexity of their chemical composition, the use of EOs requires extensive research to understand the mechanisms by which they could exert the observed therapeutic effects. In this sense, lavender and tea tree EOs have been identified as potential endocrine disruptors in prepubertal children, causing gynecomastia and premature thelarche. The ingestion of tea tree EO can cause an altered mental status, ataxia, and slurred speech. Linalool, *α*-terpineol, and 4-terpineol have shown antiandrogenic and estrogenic activity in in vitro assays [55,56]. Additionally, eugenol, cinnamal, and carvone have been shown to activate mechanisms that trigger allergic reactions, phototoxicity, and contact dermatitis [57].

The chemical composition, quantity, and nature of the phytoconstituents influence the beneficial or adverse activity of the extracts and, consequently, their therapeutic effects. For this reason, the use of essential oils must be extensively studied to ensure their safety and efficacy and to establish appropriate doses. In general, the following therapeutic properties have been established for the classes of compounds listed below: acids, C10 alcohol, aromatic aldehydes and phenols (antimicrobial and immunostimulant properties), C15 and C20 alcohols (estrogen-like activity), aldehydes (soothing, antimicrobial, and litholytic properties), coumarins and lactones (soothing and balancing properties), esters (soothing and antispasmodic properties), ketones (wound healing, litholytic, mucolytic, and soothing properties), oxides (antispasmodic and expectorant properties), phenyl methyl esters (antimicrobial and antispasmodic properties), monoterpenes (antimicrobial and cortisone-like activity), and sesquiterpenes (antihistaminic and antiallergic properties) [25].

A classification of the main therapeutic applications of EOs is described below.

### 3.1. Anxiety, Fatigue, and Sleep Disorders

Anxiety is one of the normal defense mechanisms of the human which becomes a psychiatric disorder when it appears in inappropriate contexts or its persistence is exaggerated. Anxious people manifest psychological and physiological changes generated by emotional, cognitive, behavioral, and somatic elements that trigger symptoms such as palpitations, tension, chest pain, sweating, shortness of breath, and papillary dilation as a normal response to a panic situation [31]. This disorder predominates in the female population worldwide [14]. Anxiety is a feeling of fear or inner nervousness generated when an individual faces threatening or stressful situations such as bereavement, a chronic illness, and poor quality of life, among others [58]. Anxiety disorders are one of the most significant causes of depression, which can lead to drug abuse [41]. Therefore, pharmacotherapy is limited by the delay in the therapeutic effect and the appearance of other side effects such as dependence, tolerance, and withdrawal [42].

Fatigue is a condition that leads to a decrease in the normal functions of an individual due to energy deficiency, with subsequent feelings of weakness and exhaustion, which is derived from prolonged stress. Generally, individuals with fatigue are forced to exert effort to cope with physical and cognitive activities, which leads to increased anxiety. Therefore, fatigue and anxiety are conditions that an individual experiences almost simultaneously [17]. Confusion, fatigue, and addiction are characteristic adverse effects of anxiety pharmacotherapy; for this reason, the use of non-pharmacological alternatives, including inhalation and massage aromatherapy, has emerged as one of the promising therapeutic tools due to its low cost and fewer side effects [59].

Aromatherapy is used successfully for the treatment of anxiety as it improves mood and exerts physiological effects on the patient until relaxation is achieved once essential oils are used in inhalation aromatherapy, massage, or in a particular environment. Anxiety control is one of the main objectives of aromatherapy, since this exacerbated response of the organism triggers other psychological and physiological alterations that lead to the interruption of the natural mind–body–spirit balance. For example, anxiety-induced mental anguish and sleep disturbances have been shown to induce significant delays in wound healing, leading to increased stress levels and a weakened immune system. In this case, massage with essential oils is used as a complementary therapy by nursing professionals in health systems since it is a non-invasive, inexpensive procedure that strengthens communication between patient and nurse. The volatile components of these liquid extracts have been reported to be absorbed through the skin and provide sedation, analgesia, and antispasmodic effects, in addition to improving mental status by reducing anxiety and fatigue and improving sleep quality [60].

The most used EOs are those obtained by distillation of lavender and German chamomile (*Matricaria chamomilla*, Asteraceae). These EOs exert strong anti-inflammatory and analgesic effects in burn patients [61]. Chamomile EO reduces the anxiety of patients who will undergo medical surgical procedures. This EO has also been used to relieve symptoms of depression, improve sleep quality, and reduce contractions in early labor. Its sedative and anxiolytic effects have been related to the presence of apigenin, a flavone that binds to GABA receptors to exert both biological activities [15]. Aromatherapy also is used successfully in surgeries or dental treatment, as well as for the masking of unpleasant odors from some components used in these procedures such as occurs with eugenol, an EO derived from clove (*Syzygium aromaticum*, Myrtaceae) that is part of the components of dental cement and is used as an endodontic sealer [60]. The aromatic resin of copaiba (*Copaifera officinalis*, Fabaceae) has been widely used in folk medicine for its anxiolytic, anti-inflammatory, antiparasitic, and skin-healing properties. Its main component is *β*-caryophyllene, which has been attributed to anxiolytic effects by attenuating cortisol levels in clinical trials and reducing cortisone in animals [59]. Another useful plant for the treatment of psychological disorders is padhi, also known as velvet leaf, abuta, and Pereira root (*Cissampelos pareira*, Menispermaceae). This plant is very popular in traditional Ayurveda medicine and is widely used as an antidepressant, anxiolytic, and muscle relaxant [62].

Insomnia is a common disorder that affects the quality of life of individuals because it alters their mood and induces cardiovascular and cerebrovascular diseases. Aromatherapy through the inhalation of essential oils, baths, and massages have been very useful therapeutic options to overcome this sleep disorder and maintain the integral health of affected individuals [16]. One of the frequent changes in mood is depression, which can occur due to multiple factors, including genetic, biological, psychological, and environmental factors. Ylang-ylang, lavender, chamomile, rosemary (*Salvia rosmarinus*, Lamiaceae), and cinnamon (*Cinnamomum verum*, Lauraceae) oils have been reported to help reduce this disorder and improve mood [13,40]. In older adults, lavender and chamomile are the most recommended essences due to their effectiveness in treating anxiety, depression, and stress scores [15]. Peppermint (*Mentha piperita*, Lamiaceae) is a herb used for both culinary and medicinal purposes. This herb reduces anxiety due to decreases in the corticotropin-releasing hormone, reducing cortisol secretion from the adrenal gland. Its main phytoconstituent is menthol, a natural product that acts on kappa opioid receptors, blocking the transmission of pain signals and reducing pain [46].

### 3.2. Neuroprotective Potential

Molecular and cellular changes, atrophy of certain brain areas, brain inflammation, and dementia are some neurodegenerative manifestations that lead to age-related progressive detrimental effects on the brain [34]. Neurodegeneration is associated with increased acetylcholinesterase (AChE) levels, metabolic disorders, the formation of amyloid-beta plaques, and intracellular neurofibrillary tangles. Increased AChE induces reduced levels of acetylcholine (ACh). Acetylcholine leads to synaptic alterations and cognitive dysfunction, affecting memory and learning [63]. Since progressive memory decline occurs over time, people with pathologies such as Alzheimer’s disease (AD), Lewy Body Dementia (LBD), and mild cognitive impairment (MCI) manifest difficulty in performing daily tasks. Butyrylcholinesterase (BChE) is another enzyme involved in the degradation of ACh. Inhibitors of AChE and BChE involved in the decline of ACh are the major compounds approved for the symptomatic management of these diseases [31].

EO constituents have been reported as inhibitors of both acetylcholinesterase and α-amylase, an important digestive enzyme that controls diabetes. The consumption of aromatic plants as a functional food can prevent oxidative damage caused by free radicals due to the antioxidant properties of their secondary metabolites, but also because they help to improve memory and sugar levels in the body [63]. Aroma oil is a therapy for the amelioration of dementia symptoms, olfactory disorders, and sleep quality [64]. Studies have demonstrated the beneficial effects of EOs obtained from peppermint, sage (*Salvia officinalis*, Lamiaceae), sandalwood, eucalyptus (*Eucalyptus globulus*, Myrtaceae), and rosemary due to the inhibitory activity that they exert on AChE [40]. Leaves and flowers of marsh pepper (*Polygonum hydropiper*, Polygonaceae) have been shown to inhibit AChE and BChE, but also free radical scavenging efficacy [31].

### 3.3. Relief of Dementia

Aromatherapy is effective in treating dementia and dementia-related syndromes, such as geriatric syndromes. These syndromes present episodes of anxiety, depression, agitation, delusions, misidentifications, hallucinations, wandering, and aggression. The imbalance of glutamatergic and GABAergic neurotransmissions and the overactivation of NMDA receptors have been related to the development of neuropsychiatric symptoms, common in the demented population. Dementia is characterized by progressive deficits in memory, understanding, and communication. Dementia patients have also manifested a loss of orientation, as occurs in Alzheimer’s disease [21].

Several studies indicate that aromatherapy is very useful in relieving the symptoms of dementia. This property is due to the antioxidant, neuroprotective, and AChE-inhibitory activities of some active components from EO; for example, monoterpenoids from thyme, Spanish sage, and lavender EOs, which inhibit AChE [65]. Lavender has been reported to improve balance (static and dynamic), thus reducing falls in patients with neurodegeneration. Further, the simultaneous use of rosemary and lemon essences in the morning, as well as night aromatherapy with lavender and orange essences, improves cognition, memory, intellect, and, in general, behavior and the ability to perform activities of daily living. Finally, the combination of lavender and lemon decreases irritability in patients and black pepper essential oil improves appetite and swallowing [66].

### 3.4. Pain Relief

Despite the therapeutic potential of some selective and non-selective agonists for opioid and cannabinoid receptors in the treatment of pain, these pharmacologic methods involve the appearance of side effects, among them being nausea, vomiting, allergic reactions, and impaired vital signs (respiration and heart rate, among others). Additionally, drowsiness, development of tolerance, and physical dependence have been observed in patients [50,67]. Since ancestral times, aromatic herbs such as lavender, ginger (*Zingiber officinale*, Zingiberaceae), eucalyptus, rosemary, and chamomile have been used for relief of pain and limitation in movement (for example in peripheral neuropathy, osteoarthritis, labor, and burns). Today, EOs of herbs are used by nurses in both aromatherapy and massage to decrease pain, increase patients’ ability to perform activities of daily living [18] and strengthen the immune system [68].

It has been reported that EOs access the cardiovascular system through the blood and lymphatic vessels in the epidermis. In this way, EOs constituents with analgesic properties reduce pain by inducing the release of dopamine, endorphins, norepinephrine, and serotonin. In this sense, lavender EO is well recognized for its ability to reduce pain, while ginger EO is used for its enhancement effect on joint function [68].

Osteoarthritis is a disease that affects the joints to the point of disability (movement limitations, morning stiffness, and articular tenderness) and is one of the reasons for the significant increase in the burden on health care systems. In this disease, inflammatory cytokines production exerts a pivotal role in the occurrence of pain and inflammation. For this reason, non-steroidal anti-inflammatory drugs (NSAIDs), corticosteroids, cyclooxygenases, intra-articular injection of hyaluronic acid, and joint replacement are part of conventional treatments for osteoarthritis. However, studies have shown high toxicity effects that conduce to cardiovascular problems and gastrointestinal disorders. Further, these treatments inhibit cartilage matrix synthesis and increase the damage to joint cartilage [18].

In knee osteoarthritis patients, the limited efficacy and risk of adverse effects of conventional treatments have led to the use of massage therapy using EOs as an alternative therapy to treat the pain of patients. Although the mechanism of action of massage therapy is unknown, this therapy exerts effects on muscular, nervous, and circulation systems (local and general). It has been observed that massage improves muscle pumping and stretch in soft tissues; therefore, it is very useful for the treatment of back pain. Additionally, it increases capillary circulation and tissue elasticity and improves its permeability [48]. It is postulated that it works by increasing blood supply and lymphatic flow, respectively, leading to pain reduction [69].

Diabetes mellitus (DM) is a disease that groups physiological dysfunctions characterized by hyperglycemia. This can present as an autoimmune disorder due to the deterioration of pancreatic beta cells (DM1) or due to the progressive deterioration of glucose regulation due to the combination of dysfunctional pancreatic beta cells and the insulin resistance (DM2) [70]. Of all the diabetes mellitus-induced micro- and macrovascular complications (nephropathy, neuropathy, retinopathy, coronary artery disease, peripheral arterial disease, and stroke), diabetic peripheral neuropathy (DPN) is the most common complication of both type 1 and 2 diabetes [32]. DPN is found in 11–21% of diabetic patients impacting their quality of life by neurophatic pain, foot ulcers, and lower-limb amputations, which leads to symptoms related to physiological and psychological disturbances such as sleep disturbances, declined physical activity, social isolation, depression, and anxiety, among others. Therefore, DM represents a challenge to the healthcare system, but also for individuals and society [9]. Pain control in peripheral neuropathy is vital to maintaining the quality of life of patients. Lavender, geranium (*Pelargonium* sp., Geraniaceae), rosemary, and eucalyptus EOs are some essences used in aromatherapy for their anti-inflammatory, analgesic, sedative, and anxiolytic properties [9].

Migraine is a chronic disease generated by a neurovascular dysfunction characterized by pulsatile pain (unilateral or bilateral) which can affect the quality of life of people. This condition has complex pathogenesis; therefore, its treatment is complicated. Aromatherapy is an alternative strategy used by patients for alleviation of their symptoms because acts against main migraine symptoms (pain, photophobia, nausea, and vomiting). Plants such as Chinese aconite (*Aconitum carmichaeli*, Ranunculaceae), Canada mint (*Mentha canadensis*, Lamiaceae), black Pepper (*Piper nigrum*, Piperaceae), Safflower (*Carthamus tinctorius*, Asteraceae), frankincense (*Boswellia sacra*, Burseraceae), myrrh gum (*Commiphora myrrha*, Burseraceae), sambong (*Blumea balsamifera*, Asteraceae), clove, melon (*Cucumis melo*, Cucurbitaceae), and ginger are some species recognized as antimigraine herbs [20].

Psychological alterations such as stress and anxiety experienced by a woman during labor are associated with the activation of the sympathetic nervous system and the secretion of hormones and neurotransmitters such as cortisol, epinephrine, and norepinephrine [71]. This leads to a decrease in uterine contractions and causes prolonged labor. The perceived pain is due to cervical dilation, contraction of the pelvic floor/perineal muscles, and uterine extension. Under this scenario, the aromatic plants used in aromatherapy play a fundamental role in reducing both anxiety and the perception of pain.

Damask rose is a plant with beneficial therapeutic effects for women in labor. These effects are related to its stimulating properties on the limbic system, the reduction of cortisol, the increase of serotonin levels and anti-inflammatory, analgesics, and antispasmodics effects of its secondary metabolites present in EO [72,73].

The analgesic effects of aromatherapy are related to the following factors: (a) The essential oil reaches the pleasure memory sites located in the brain; (b) volatile components with analgesic properties modulate the activity of the neurotransmitters dopamine and serotonin, as well as norepinephrine receptor sites in the brain; (c) the interaction of touch with the sensory fibers of the skin occurs effectively; and (d) the absorption of EOs into the bloodstream is increased by massage [49].

The analgesic properties of damask rose and orange are recognized due to their high content of flavonoids. Damask rose is amply used for postoperative pain relief induced by abdominal, cholecystectomy, urogenital, thorax, and facial surgeries, while orange is used for moderate-to-severe knee pain relief. Hypotheses indicate that aromatic compounds from damask rosa and orange stimulate the release of endorphins generating pleasure effects and well-being with subsequently decreasing pain severity [74]. *Eucalyptus citriodora* (Myrtaceae) and lavender are two aromatic plants recognized for their anti-inflammatory, neuroprotective, analgesic, and muscle-relaxing properties. Sour orange (*Citrus aurantium*, Rutaceae) is a plant that exerts sedative effects and also induces sleep and appetite and alleviates heart palpitation [50]. Other plants well known for their analgesic, antineuralgic, anti-inflammatory, antinociceptive, and muscle-relaxing properties are rose-scented geranium (*Pelargonium graveolens*, Geraniaceae), rosemary, and chamomile (*Chamaemelum recutita*, Asteraceae) [49].

### 3.5. Relief of Symptoms in Cardiovascular Diseases

Cardiovascular diseases (CVDs) are one of the leading causes of morbidity and mortality worldwide, and according to the World Health Organization (WHO), these represent 31% of annual global deaths [75]. CVDs involve those pathologies that compromise the integrity of the heart and blood vessels such as atherosclerosis, hyperlipidemia, systolic hypertension, venous and cerebral insufficiencies, angina pectoris, and congestive heart failure. Although conventional medicine has made great advances to treat these diseases, the side effects and high cost of medications lead patients and health professionals to use other therapeutic alternatives, one of which is the use of traditional knowledge of aromatic plants. For centuries, plants have been used to prevent or alleviate the symptoms of cardiovascular diseases. Even today, this therapeutic approach is a medicinal alternative in health care systems. Although the use of plant extracts or inhalation aromatherapy to relieve symptoms is based on folk medicine, in vitro and in vivo studies are still important to demonstrate their efficacy and safety. Some species that have been used widely in folk medicine have been useful in relieving psychologic symptoms such as stress, depression, and anxiety, as well as physiologic symptoms, among them systolic/diastolic BP, heart rate, and CVD-associated conditions such as oxidative stress, inflammation, hypertension, and hyperlipidemia [76].

Popular knowledge describes garlic (*Allium sativum*, Amaryllidaceae) as one of the most useful plants to treat cardiovascular diseases and conditions associated with them (oxidative stress, hypertension, and others) since it has the property of decreasing total cholesterol, LDL, and the content of lipids in arterial cells; therefore, it is useful in controlling atherosclerosis and hyperlipidemia [33]. Rosemary is another aromatic plant as common as garlic for its culinary properties. Studies have shown that hydroalcoholic extract from rosemary increases cerebral blood flow, making it useful for treating brain failure associated with ischemic stroke. Its beneficial properties extend to reducing the expression of proinflammatory enzymes and mediators, such as inducible NO synthase (iNOS) and cyclooxygenase-2 (COX-2) [77]. Other useful plants to treat CVD are lemon and lavender. Lemon has been shown to exert effects on blood pressure and electrocardiogram changes in patients with acute myocardial infarction [76], while lavender reduces physiological parameters, mean arterial pressure, systolic and diastolic blood pressure, and heart rate [76].

Excessive stress and other psychologic disorders lead to significant alterations of the sympathetic nervous system, generating increased blood pressure or changes in heart rate. Therefore, psychological alterations such as anxiety, depression, and sleep disorders are the most common in CVD patients. To treat these complications, as well as for relieving pain and nausea, inhalation aromatherapy with chamomile, damask rose, ginger, lavender, lemon balm (*Melissa officinalis*, Lamiaceae), tangerine (*Citrus reticulata*, Rutaceae), and lemon EOs is a strategy in the cardiac care units [78]. From this list, lemon balm is effective in preventing cardiac abnormalities derived from benign palpitations manifested in anxious patients. Further, it enhances the activity of gamma-aminobutyric acid (GABA), so it is useful to treat psychological disorders. It is well known that this plant relieves muscle spasms, sleep disorders, and reduces hypertension and blood glucose levels [79].

### 3.6. Relief of Complications in Cancer Patients

Cancer is a disease considered the 2nd cause of death worldwide next to cardiovascular diseases [80], whose therapeutic options are aimed at increasing patients’ quality of life. Despite the advances in cancer treatment through surgery, chemotherapy, and radiotherapy, as well as the use of medical interventions such as hormones, biological therapies, and immunotherapy, cancer patients do not have a definitive treatment and suffer the consequences of the negative side effects that occur with these therapeutic options such as dependency and tolerance. This disease changes many aspects of life, causing discomfort in patients. This way, inhalation aromatherapy and massages with aromatic essences offer the cancer patient relief from common symptoms such as sleep disorders, pain, depression, anxiety, fatigue, nausea, and vomiting in an effective, safe, and economical way, considering their organic nature, low risk, and low costs, but rigorous studies are needed to prove their efficacy [45,81].

### 3.7. Relief of Nausea and Vomiting

Electrolyte imbalance and risk of aspiration and dehydration are some of the complications presented in episodes of nausea and vomiting. To treat these discomforts, aromatherapy is an effective strategy, not only for cancer patients but also for pregnant women and postoperative patients. This discipline has been used as an alternative to antiemetic drugs since these have adverse effects associated that can result in patient dissatisfaction, as well as increased lengths of stay and hospital costs [35,82]. It has been reported that peppermint, ginger, chamomilla, or cardamom (*Elettaria cardamomum*, Zingiberaceae) are some plants used to relieve nausea and vomiting in oncologic patients [83]. Furthermore, it has been reported that combinatorial inhalation aromatherapy with peppermint and lemon exerts antiemetic effects in pregnancy [84], while peppermint EO inhalation is employed to relieve postoperative nausea and vomiting (PONV) in patients undergoing cardiac surgery [85].

### 3.8. Relief of Respiratory Tract Infections

In recent years, there has been a growing interest in the use of aromatic plants for phytotherapeutic purposes and EOs for inhalation aromatherapy as a complementary and alternative medicine for the treatment of respiratory tract infections. This is because respiratory tract infections are causing high mortality rates globally and new treatments are needed to combat them [86]. The typical bacteria associated with respiratory infections include *Streptococcus pneumoniae*, *Haemophilus influenzae*, and *Staphylococcus aureus* [87], while viral respiratory infections are due to the influenza virus and adenoviruses, mainly [87]. One of the microorganisms that most commonly affects the respiratory tract is *S. pneumonia*.

*Streptococcus pneumoniae* is the causal agent of community-acquired pneumonia, bacterial sinusitis, bacterial meningitis, and otitis media [88]. The development of new antibiotics and reinforcing the immune system by actively modulating host immunity or interfering with pneumococcal virulence factors are some therapeutic strategies for effectively treating bacterial infections in the respiratory tract. However, the high costs of the research and development of these therapeutic alternatives have led to the exploration of medicinal plants as a promising solution for treating these infections [37].

Today, the treatment of respiratory tract infections includes conventional antibiotics and vaccinations as preventive measures. However, these infections involve various microorganisms, which have specific structural and biochemical characteristics with certain pathogenicities and resistances [89]. Therefore, inappropriate prescription practices could induce microbial resistance to antibiotics. In this circumstance, the use of phytotherapy is a viable option.

It has been reported that the phytotherapy used in infusions, decoctions, tinctures, and phytochemicals by health professionals and practitioners of traditional medicine has allowed the relief of the symptoms of nasal discomfort and pulmonary congestion caused by infections related to bacteria and viruses, thus increasing the scientific interest in aromatic herbs to treat upper and lower respiratory tract infections [43].

**Figure 4:**
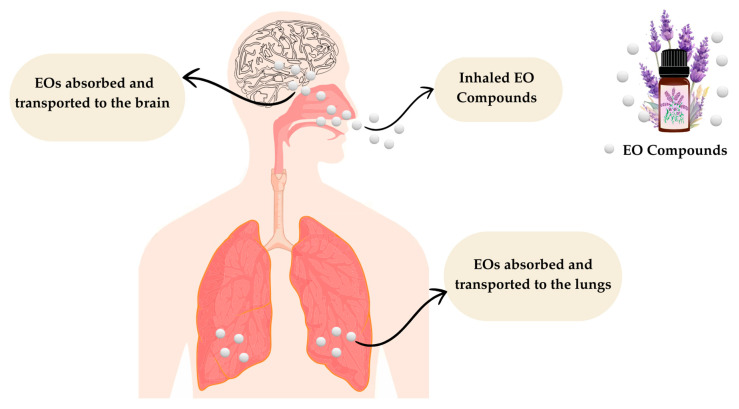
Routes of absorption of inhalation aromatherapy. Adapted from Leigh-de Rapper and van Vuuren [87].

The respiratory tract has natural barriers that prevent infections caused by pathogens (bacteria or viruses). Among these barriers are nasal hair, phagocytic and mucociliary cells, and residual microbiota. Under certain conditions, pathogens can overcome these barriers and initiate an infectious process that leads to the release of histamine, giving rise to an inflammatory response characterized by pain, mucus production, and coughing. EO use in inhalation aromatherapy is a complementary strategy that is used to effectively reduce the symptoms generated. Therefore, in the presence of an infectious process, essential oils relieve not only the mood but also act at the level of the respiratory tract, since after inhalation, the volatile components can disperse both in the upper respiratory tract and lower respiratory tract, exerting therapeutic benefits on an affected individual [87]. The main routes of absorption of inhalation aromatherapy are shown in Figure 4.

Studies have shown that the combination of pine, peppermint, and lemon is effective for throat infections and nasal congestion with little or no adverse events [87]. Anise (*Pimpinella anisum*, Apiaceae) and bitter fennel fruit (*Foeniculi amari* fructus aetheroleum, Apiaceae) are used as an expectorant in cough associated with colds; eucalyptus is used in coughs, colds, and bronchitis; peppermint relieves digestive disorders (flatulence and irritable bowel syndrome, among others) and symptomatically treats coughs and colds; tea tree (*Melaleuca alternifolia*, Myrtaceae) is useful in colds, influenza, and bronchitis; and thyme (*Thymus vulgaris*, Lamiaceae) is used in bronchial catarrh and supportive treatment of pertussis [90].

### 3.9. Relief of Skin Infections

Acne is a chronic and inflammatory disease of the skin, specifically of the pilosebaceous complex, highly common among teenagers, but also can affect individuals through adulthood [91]. Acne induces primary lesions (blackheads, papules, and pustules) and secondary lesions (erythema, postinflammatory hyperpigmentation, and scarring). These lesions can exert a negative psychosocial impact since acne appears on highly exposed parts of the body such as the face, trunk, and back where sebaceous follicles predominate, altering the integrity of the skin to give rise to an unpleasant appearance.

Recent studies have permitted updating the knowledge of the etiopathogenesis of acne, which has made it possible to establish a series of guidelines for the potential treatments of this disease, among them antioxidants, vitamin D analogs, systemic and topical antiandrogens, immunotherapy, monoclonal antibodies, topical retinoic and metabolism-blocking agents, antimicrobial peptides, acetylcholine inhibitors, interleukin-1β (IL-1β) inhibitors, phosphodiesterase inhibitors, insulin-like growth factor (IGF)-1 inhibitors, peroxisome proliferator-activated receptor (PPAR) modulators, dapsone, and melanocortin receptor antagonists [92].

Despite these findings, the use of plant extracts and essential oils continues to be part of the beauty and skin-care routine, especially due to the beneficial properties that they provide, exerting a direct effect on acne or on the psychology of the individual. In this sense, eucalyptus, peppermint, lavender, rose, sandalwood, tea tree, copaiba, vetiver, calamus (*Acorus calamus*, Acoraceae), benzoin (*Styrax benzoin*, Styracaceae), and frankincense are listed among the most recognized anti-acne aromatic plants [25].

### 3.10. Relief of Menopause Symptom

Estrogen deficiency in a woman leads to menopause, a stage marked by the absence of menstruation. Many women experience problems with sexual function, mood disorders, sleep problems, hot flashes, an increased risk of osteoporosis, vascular diseases, weight gain, and cognitive impairment [93]. As a consequence of these alterations, insomnia, irritability, and depression are a constant in women at this stage of their life cycle.

It has been observed that from perimenopause, women use complementary and alternative medicine to benefit their health. One strategy consists of the inclusion of herbal supplements to increase sexual desire, improve sleep habits, and relieve psychological and physiological symptoms. In this sense, lavender has been widely used for its antioxidant, sedative, anxiolytic, and antidepressant activity and as a preventive of sleep disorders, all of which are associated with its chemical composition: tannins, flavonoids, linalool, linalyl acetate, and coumarins, among others [36,94]. Fennel has been reported as an aromatic plant effective to relieve vasomotor symptoms, sexual function, sexual satisfaction, and sleep disturbance [95]. Combinatory aromatherapy with lavender, fennel, rose, and geranium improves sexual dysfunction but does not induce changes in serum estrogen levels [96].

### 3.11. Relief of Restless Leg Syndrome

Restless leg syndrome (RLS) is a common sensorimotor disorder where patients feel the urge to perform periodic leg movements, especially during resting wakefulness or before sleeping. This syndrome affects the quality of life and mood of patients because it induces sleep disorders. RLS can appear alone or with comorbidities such as iron deficiency, renal, cardiovascular, rheumatological, neurological, and respiratory disorders, or diabetes mellitus since they are closely related to its exacerbation [97]. It has been reported that 30–50% of patients with end-stage renal disease (ESRD), especially patients undergoing hemodialysis, suffer from RLS. In these patients, orange and lavender EOs used in massage therapy have been related to the stimulation of the cerebral cortex and an enhancement in dopamine production, reducing tendinous and muscle elasticity problems and providing relief from the symptoms of RLS [19,98].

## 4. Advances in Research on Aromatic Plants in Complementary and Alternative Medicine

Results of the review of aromatic plants as natural complementary and alternative medicine are listed in Table 1. In general, the table summarizes the main therapeutic benefits of 85 species of plants belonging to 37 families which can be very useful for achieving the mind–body–spirit balance in the patients of the different healthcare units. It is shown that the plant families Apiaceae, Lamiaceae, and Rutaceae gather the largest number of promising species, while lavender, bergamot, geranium, rosemary, and peppermint are the species that have shown a wide spectrum of bioactivities.

## 5. Challenges in Evaluating Aromatherapy-Essential Oils

AEOs’ assessment in CAM faces significant challenges, particularly concerning insufficient clinical trials, the absence of standardized dosages, and long-term effects. Current literature indicates that while EOs, such as lavender, may exhibit multiple benefits, including an anxiolytic effect, the evidence supporting their efficacy remains inconclusive. A review of randomized controlled trials highlighted positive results in reducing anxiety symptoms but emphasized the need to improve methodological quality in future studies to validate these findings [109].

To enhance the evaluation of CAM therapies, it is essential to employ rigorous methodologies. Key approaches include conducting systematic reviews and meta-analyses of randomized, placebo-controlled trials, utilizing statistical techniques, including the random effects model and the inverse variance method. These methodologies provide a robust framework for synthesizing evidence, allowing for a comprehensive assessment of the efficacy and safety of CAM interventions [109,110,111].

Current evidence indicates that aromatherapy can provide a variety of benefits. However, the need for standardized application methods and dosages is necessary to make reliable comparisons between studies. In this sense, establishing standardized practices will ensure that people with CAM therapies receive safe and effective treatment, fostering greater confidence and acceptance of aromatherapy in clinical settings through the search for holistic health solutions.

## 6. Ethical Considerations in Promoting Complementary and Alternative Medicine Therapies with Limited Clinical Evidence

Promoting CAM therapies, which focus on holistic treatment of the mind, body, and spirit, raises ethical issues, particularly for vulnerable groups. Health should be understood as comprehensive well-being, necessitating the integration of evidence-based therapies to address the needs of people [112]. Over the past decades, ethical guidelines for research involving human subjects have evolved, emphasizing the importance of participant welfare, informed consent, and the equitable distribution of risks and benefits [113].

Vulnerable groups, including low-income individuals or racial minorities, i.e., marginalized communities, which often encounter barriers to healthcare, including discrimination and limited access to effective treatments, often turn to CAM therapies due to dissatisfaction with conventional treatments or a desire for more “natural” options [114]. However, in the absence of strong clinical evidence to support these therapies, there is a risk of subjecting people to ineffective or even harmful treatments with no demonstrable benefits.

Ethical principles such as nonmaleficence and beneficence demand that researchers prioritize therapies that offer significant social or scientific value, ensuring that participants are not placed at risk for trivial outcomes [115]. Therefore, establishing clear ethical standards for CAM research is pivotal to protect vulnerable populations and ensuring that their participation contributes to meaningful advancements in health care.

## 7. Conclusions and Future Perspectives

The increasing use of aromatic plants in CAM highlights their important medicinal potential. Many of these plants have well-documented therapeutic applications; however, further research is essential to explore their therapeutic properties, potential toxic effects, and mechanisms of action. The increasing popularity of inhalation aromatherapy and massage with EOs in healthcare settings requires expanded in vitro and in vivo studies to confirm their beneficial effects and provide rigorous testing of therapeutic strategies.

The appeal of EOs is often linked to the perception that natural products have fewer side effects than conventional treatments. This belief may introduce selection bias, as people who choose essential oils may have positive expectations that influence reported outcomes. Furthermore, the pleasurable sensations associated with aromatherapy may contribute to its efficacy, raising questions about potential affective or placebo effects that could bias research results.

To address these biases, future research should prioritize comprehensive investigations into the therapeutic properties of aromatic plants, while employing rigorous study designs such as randomized controlled trials. Broader knowledge will lead to integrating these natural practices into the healthcare system, ultimately improving patient outcomes and expanding the reach of CAM.

## Figures and Tables

**Table 1:** Aromatic plants used in complementary and alternative medicine for managing physiological and psychological symptoms.

Families	Scientific Name	Common Name	Main Benefits	Reference
Acanthaceae	*Andrographis paniculate* (Burm.f.) Wall. ex Nees	Green chireta	↓ Systolic hypertension	[75]
Acoraceae	*Acorus calamus* L.	Aromatic calamus	↓ Acne	[25]
Amaryllidaceae	*Allium sativum* L.	Garlic	↓ Atherosclerosis, ↓ hyperlipidemia, ↓ systolic hypertension	[75]
	*Narcissus poeticus* L.	Poet’s narcissi	↓ Dementia, ↓ neurodegenerative manifestations	[99]
Annonaceae	*Cananga odorata* (Lam.) Hook.f. and Thomson	Ylang-ylang	↓ Mood disorders, ↓ anxiety, ↓ neurophatic pain	[100]
Apiaceae	*Angelica dahurica* (Hoffm.) Benth. and Hook.f. ex Franch. and Sav.	Chinese angelica	↓ Migraine	[20]
	*Apium graveolens* L.	Celery	↓ Systolic hypertension	[75]
	*Conioselinum anthriscoides* ’Chuanxiong’	Hemlock parsley	↓ Migraine	[20]
	*Coriandrum sativum var. microcarpum* DC.	Cilantro or Chinese parsley	↓ Depression, ↓ anxiety, antioxidant	[31]
	*Foeniculum vulgare.* Mill.	Nafae	↓ Anxiety	[41]
	*Matricaria recutita* L.	Chamomile	↓ anxiety	[41,61]
Apocinaceae	*Rauvolfia serpentina.* Benth. ex Kurz	Indian Snakeroot	↓ Systolic hypertension	[75]
Araliaceae	*Panax* sp. (ginseng)	Korean ginseng	↓ Systolic hypertension	[75]
	*Panax notoginseng* (Burkill) F.H.Chen	Chinese ginseng	↓ Angina pectoris	[75]
Aristolochiaceae	*Asarum sieboldii* Miq.	Wild ginger	↓ Migraine	[20]
Asteraceae	*Anthemis nobilis* L.	Roman Chamomile	↓ Anxiety	[41]
	*Bidens pilosa* L.	Beggar’s Tick	↓ Systolic hypertension	[75]
	*Blumea balsamifera* DC.	Sambong	↓ Migraine	[20]
	*Carthamus tinctorius* L.	Safflower	↓ Migraine	[20]
Asparagaceae	*Ruscus aculeatus* L.	Butcher’s broom	↓ Venous insufficiency	[75]
Burseraceae	*Boswellia sacra* Flück. *Boswellia frereana* Birdw.	Frankincense Elemi frankincense	↓ anxiety, ↓ stress ↓ anxiety, ↓ stress	[40] [40]
	*Commiphora myrrha* (T.Nees) Engl.	Myrrh	↓ Migraine	[20]
	*Commiphora mukul* (Hook. ex Stocks) Engl.	Guggul	↓ Atherosclerosis, ↓ hyperlipidemia	[75]
Cistaceae	*Cistus creticus* L.	Pink rock-rose	↓ Dementia, ↓ neurodegenerative manifestations	[31]
	*Cistus libanotis* L.	False rosemary, rock-rose of Lebanon	↓ Dementia, ↓ neurodegenerative manifestations	[31]
	*Cistus salviifolius* L.	sage-leaved rock-rose	↓ Dementia, ↓ neurodegenerative manifestations	[31]
Cucurbitaceae	*Cucumis melo* L.	Melon	↓ Migraine	[20]
Fabaceae	*Copaifera officinalis* L.	Copaiba balsam	↓ Bacterial infections	[101]
Geraniaceae	*Pelargonium* spp.	Geraniums	↓ Anxiety, ↓ labor pain	[41]
	*Pelargonium graveolens* L’Hér.	Geraniums	↓ Bacterial infection, ↓ fungal infection, ↓ blood congestion, ↓ toxins, ↓ phlebitis, ↓ hemorrhoid, ↓ indigestion, ↓ infertility ↓ anxiety, ↓ anger, ↓ agitation, ↓ depression, ↓ menopausal symptoms, ↓ throat infections, ↑ wound healing, ↑ blood circulation, ↑ lymphatic system, astringent, hemostatic, antioxidant, supportive therapy in uterine and breast cancer and for blood disorder diabetes	[29,102]
Ginkgoaceae	*Ginkgo biloba* L.	Maidenhair tree	↓ Cerebral insufficiency	[75]
Iridaceae	*Crocus sativus* L.	Saffron crocus	↓ Systolic hypertension	[75]
Lamiaceae	*Hyssopus officinalis* L.	Hyssop	↓ Allergy, sedative	[41]
	*Lavandula angustifolia* Moench	English lavender	↓ Post-operative pain, ↓ labor pain, ↓ anxiety, ↓ stress, ↓ depression, ↑ sleep, ↓ stress, ↓ headache, ↓ skin alterations, ↓ fatigue, ↑ immune system, ↑ tissue repair, ↓ behavioral alterations, ↓ dementia in Alzheimer’s disease, ↓ migraine, ↓ acne, narcotic, sedative, soothing	[12,25,28,40,41,47,102]
	*Lavandula latifolia* Medik.	Broadleaved lavender	↓ Headache, ↓ stress, ↓ nose infections, ↓ throat infections, ↓ mood disorders, ↓ stress, ↓ depression, ↓ anxiety, ↓ migraine, ↓ chronic pain, ↓ behavioral disturbances in dementia, ↑ relaxation	[11,41]
	*Lavandula officinalis* Chaix	English lavender	↓ Anxiety, ↓ stress, ↓ depression	[12]
	*Melissa officinalis* L.	Lemon balm	↓ Anxiety, ↓ acute stress, ↓ pain intensity, ↓ hemodynamic parameters in acute coronary syndrome, ↑ relaxation, ↑ immune support	[41,79]
	*Mentha canadensis* L.	Canada mint	↓ Migraine	[20]
	*Mentha longifolia* (L.) L.	Horsemint	↓ Systolic hypertension	[75]
	*Mentha piperita* L.	Peppermint	↓ Mental fatigue, ↑ memory, ↑ cognition, ↓ cold and flu symptoms, ↓ migraine, ↓ labor pain, ↓ exhaustion, ↓ nausea, ↓ dementia, ↓ agitation, ↓ acne	[25,40,41]
	*Ocimum basilicum* L.	Common Basil	↑ Sedative, ↓ anxiety	[28,41]
	*Pogostemon cablin* (Blanco) Benth.	Patchouli	↓ Blemishes, ↓ wrinkles	[28]
	*Salvia sclarea* L.	Clary sage	↓ Stress, sleep-promoting, ↓ anxiety, ↓ menstrual cycle alteration, ↓ blood pressure, ↓ panic, ↓ abdominal tension, ↓ muscle cramps, ↓ cortisol, ↓ sebum production, ↑ memory, aphrodisiac activity	[28,40,41,102]
	*Salvia miltiorrhiza* Bunge	Danshen	↓ Systolic hypertension, ↓ angina pectoris	[75]
	*Rosmarinus officinalis* L.	Rosemary	↓ Anxiety, ↑ mood, ↑ cognitive function, ↓ localized blood flow in the brain, ↓ cerebral insufficiency	[40,75]
Lauraceae	*Cinnamomum verum* J.Presl	Cinnamon	↓ Depression, ↓ anxiety	[40]
	*Litsea cubeba* (Lour.) Pers.	Mountain pepper	↓ Cognition-associated discomforts, ↓ mood alterations	[8]
Malvaceae	*Abelmoschus moschatus* Medik.	Musk mallow	↓ Migraine	[20]
	*Hibiscus sabdariffa* L.	Roselle	↓ Systolic hypertension	[75]
Menispermaceae	*Cissampelos pareira* L.	Padhi, velvet leaf, abuta, Pereira root	↓ Depression, ↓ anxiety, ↓ muscle contraction, ↓ acne	[62]
Myrtaceae	*Eucalyptus* sp.	Eucalyptus	↓ Throat infections, ↓ coughs, ↓ bronchitis, ↓ sinusitis, ↓ asthma, ↓ rheumatoid arthritis symptoms, ↓ nasal congestion, ↓ persisting mucus, ↓ catarrh, ↓ asthma, ↓ migraine, ↓ acne	[25,41,102]
	*Eucalyptus globulus* Labill.	Eucalyptus	↓ Pain, ↓ stress, ↓ anxiety	[40]
	*Melaleuca alternifolia* (Maiden and Betche) Cheel	Tea tree	↑ Antiseptic properties (ventilation and cleanliness), ↓ acne	[10,25]
	*Myrtus communis* Blanco	Myrtle	↓ Whooping cough, ↓ bronchitis, ↓ respiratory infections	[41]
	*Syzygium aromaticum* L. Merr. and L.M.Perry	Clove	↓ Pain, ↓ migraine	[20,40]
Oleaceae	*Jasminum officinale* L.	Common jasmine	↓ Labor pain, ↑ relaxation	[41]
	*Jasminum sambac* (L.) Aiton	Arabian Jasmine	↓ Neurodegenerative disorders, ↓ dementia, ↓ anxiety, ↓ depression, ↓ cognitive hypofunction epilepsy, ↓ convulsions	[31]
	*Osmanthus fragrans* (Thunb.) Siebold	Fragrant olive	↓ Anxiety	[38]
Piperaceae	*Piper longum* L.	Indian Long Pepper	↓ Migraine	[20]
	*Piper nigrum* L.	Black Pepper	↓ Neurodegenerative disorders	[31]
Plantaginaceae	*Digitalis purpurea* L.	Foxglove	↓ Congestive heart failure	[75]
Poaceae	*Vetiveria zizanioides* (L.) Nash	Vetiver	↓ Anxiety	[41]
	*Cymbopogon citratus* (DC.) Stapf	Lemongrass	↓ Anxiety, ↓ systolic hypertension	[41,75]
Polygonaceae	*Polygonum hydropiper* L.	Marshpepper knotweed	↓ Neurodegenerative disorders	[31]
Ranunculaceae	*Aconitum carmichaeli* Debeaux	Chinese aconite	↓ Migraine	[20]
	*Aconitum kusnezoffii* Rchb.	Chinese aconite	↓ Migraine	[20]
	*Coptis chinensis* Franch.	Huang Lian	↓ Atherosclerosis, ↓ hyperlipidemia, ↓ congestive heart failure	[75]
	*Nigella sativa* L.	Black cumin	↓ Systolic hypertension	[75]
Rosaceae	*Crataegus oxyacantha* L.	Hawthorn	↓ Systolic hypertension, ↓ angina pectoris	[75]
	*Rosa damascena* Herrm.	Damask rose	↓ Anxiety, ↓ pain and anxiety in the first stage of labor, ↓ sleep problems, physiological and psychological relaxation, relaxant, antitussive, hypnotic, antioxidant, antibacterial and anti-diabetic effects	[31,41,72,103]
Rubiaceae	*Uncaria rhynchophylla* (Miq.) Miq.	Cat’s claw	↓ Systolic hypertension	[75]
Rutaceae	*Citrus aurantium var. Amara*	Neroli	↓ Sleep problems, ↓ sore throat, ↓ labor pain, ↓ anxiety, ↓ stress, sedative, relaxant, soothing, ↑ positive mood	[41,104]
	*Citrus bergamia* Risso	Bergamot	↓ Mood alteration, ↓ anxiety, ↓ pain, ↓ stress, ↓ inflammation, ↓ sleep disorders, ↓ dementia, ↓ depression, ↓ migraine, ↓ chronic pain, ↓ behavioral disturbances in dementia	[11,105]
	*Citrus limon* (L.) Osbeck	Lemon	↓ Anxiety, ↓ depression	[41]
	*Citrus paradisi* Macfad.	Pomelo or grapefruit	↓ Appetite	[38]
	*Citrus sinensis* (L.) Osbeck	Orange	↓ Anxiety, ↓ migraine, ↓ labor pain	[41]
Sapindaceae	*Aesculus hippocastanum* L.	Horsechestnut	↓ Venous insufficiency	[75]
Santalaceae	*Santalum album* L.	East Indian sandalwood	↓ Anxiety, ↓ Acne, ↓ bacterial infections, ↓ inflammation, sedative, hypnotic	[25,41]
	*Santalum paniculatum* Hook. and Arn.	Mountain sandalwood, Hawaiian sandalwood	Antioxidant	[40]
Styracaceae	*Styrax benzoin* Dryand.	benzoin	↓ Acne, ↓ bacterial infections, ↓ inflammation	[25]
Theaceae	*Camellia sinensis* (L.) Kuntze	Green tea	↓ Systolic hypertension	[75]
Urticaceae	*Urticaria dioica* (K.Brandegee) Doweld	Stinging nettle	↓ Systolic hypertension	[75]
Zingiberaceae	*Curcuma longa* L.	Turmeric	↓ Mood alterations, ↓ stress, ↓ depression, ↓ anxiety, ↓ migraine, ↓ chronic pain, ↓ behavioral disturbances in dementia	[11]
	*Zingiber officinale* Roscoe	Ginger	↓ Nausea and vomiting, ↓ migraine, ↓ appetite disorders, ↓ flatulent, ↓ hemorrhoid, ↓ post-partum pain	[20,106,107,108]

↓ Decrease, ↑ Increase.
